# A Method for Oscillation Errors Restriction of SINS Based on Forecasted Time Series

**DOI:** 10.3390/s150717433

**Published:** 2015-07-17

**Authors:** Lin Zhao, Jiushun Li, Jianhua Cheng, Chun Jia, Qiufan Wang

**Affiliations:** 1 College of Automation, Harbin Engineering University, Harbin 150001, China; E-Mails: lijiushun2015@163.com (J.L.); ins_cheng@163.com (J.C.); jiachuntzjz@163.com (C.J.); 2 Beijing Institute of Automatic Control Equipment, Beijing 100074, China; E-Mail: wangqiufan083623@sina.com

**Keywords:** SINS, oscillation errors, forecasted time series, oscillation errors restriction

## Abstract

Continuity, real-time, and accuracy are the key technical indexes of evaluating comprehensive performance of a strapdown inertial navigation system (SINS). However, Schuler, Foucault, and Earth periodic oscillation errors significantly cut down the real-time accuracy of SINS. A method for oscillation error restriction of SINS based on forecasted time series is proposed by analyzing the characteristics of periodic oscillation errors. The innovative method gains multiple sets of navigation solutions with different phase delays in virtue of the forecasted time series acquired through the measurement data of the inertial measurement unit (IMU). With the help of curve-fitting based on least square method, the forecasted time series is obtained while distinguishing and removing small angular motion interference in the process of initial alignment. Finally, the periodic oscillation errors are restricted on account of the principle of eliminating the periodic oscillation signal with a half-wave delay by mean value. Simulation and test results show that the method has good performance in restricting the Schuler, Foucault, and Earth oscillation errors of SINS.

## 1. Introduction

Owing to its autonomy, continuity, and comprehensiveness of navigation, strapdown inertial navigation system (SINS) plays an irreplaceable role in military and civil navigation fields and it is used as the core navigation system in navigation applications [1,2,3,4].

In addition to that errors of inertial navigation systems are accumulated over time, the error sources (such as the initial alignment errors, gyros and accelerometer measurement errors, *etc.*) cause inertial navigation system to have a variety of errors which contain Schuler, Foucault, and Earth periodic oscillation errors. These oscillation errors greatly reduce the precision of inertial navigation system [5,6]. For example, Schuler and Foucault periodic oscillation errors have a negative influence on the precision of horizontal velocity and attitude, while Earth periodic oscillation errors reduce the accuracy of azimuth and position, which is adverse to navigation systems required to provide the high-precision navigation and positioning information. iXBlue is a leading global provider of innovative solutions and services for navigation, positioning, and imaging. Civil and defense customers rely on their systems, operations, and services for the challenges faced at sea, on land, in the air or in space. iXBlue presented navy testing results of the iXBlue MARINS FOG INS in 2014 and parts of the results are shown as follow [7].

**Figure 1:**
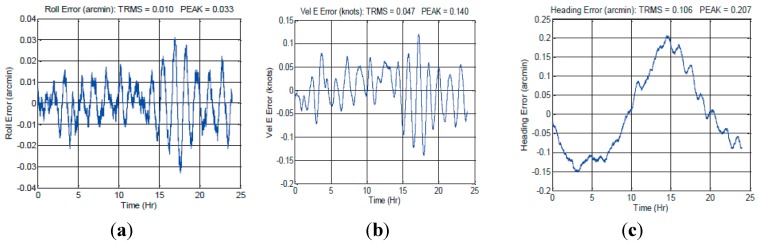
Navy testing results of the iXBlue MARINS FOG INS: (**a**) Roll error; (**b**) East velocity error; (**c**) Heading error.

As shown in Figure 1, there are oscillation errors in the system errors, obviously, which reduces the accuracy of SINS. Therefore, damping technology and integrated navigation technology are often used to restrain the oscillation errors of inertial navigation system [8,9]. Although there are some defects in the application of damping technology and integrated navigation technology, these two technologies are still used as the main tools of restraining the oscillation errors [7].

The inertial navigation system with a damping network is called damping inertial navigation system. Based on the classical control theory, the traditional damping method is to design the damping network that is in series with the system, which can suppress the oscillation errors of the inertial navigation system effectively. However, the added damping network destroys the free interference condition of the inertial navigation system and brings dynamic errors, caused by maneuvers, to the damping inertial navigation system, reducing the accuracy of the inertial navigation system [10,11]. The damping inertial navigation system is confined to the condition that the carrier is with no acceleration or small acceleration. Nevertheless, the carrier cannot always be in its state of rest or of uniform motion in a straight line, so the inertial navigation system needs to frequently switch between the damping and the free damping states [12,13,14,15,16]. To reduce the overshoot in states switch, instantaneous velocity of SINS at the switch moment is compensated to the damping network in paper [17], which can reduce the velocity error overshoot and its steady-state error at the same time. In order to weaken the negative effect of the damping network on the inertial navigation system, the damping coefficient of the damping network is, relatively, smaller [7]. The damping inertial navigation system can also be actualized by using Kalman filtering technology [18,19]. American mainstream marine inertial navigation systems, such as AN/WSN-7B and MK49, have employed the damping method based on Kalman filtering technology and the manual switch between the traditional damping method and Kalman damping method is viable. However, due to the defect of the design principle for damping inertial navigation systems, either the traditional damping method or Kalman damping method is unable to be used in the high-maneuvering condition and suppress the oscillation errors continuously.

Integrated navigation system is a navigation system that combines two or more than two navigation subsystems in an appropriate way to achieve some purpose, such as to improve the accuracy of the system [20,21,22,23]. The emergence of various filtering algorithms provides a good theoretical and mathematical tool for the integrated navigation system [24,25,26]. Owing to this, the integrated navigation system can effectively restrain the errors of the inertial navigation system. Integrated navigation systems, especially the GPS/INS integrated navigation system, has been widely used in various fields [27,28,29,30,31]. Although the integrated navigation system can improve the precision of the inertial navigation system, help from external information makes inertial navigation system lose its autonomy, which is an inevitable shortcoming of integrated navigation. 

Based on the analysis of inertial navigation system errors and the errors’ propagation mechanism, this paper proposes a method for oscillation error restriction of SINS based on forecasted time series. In the view of the math, making use of the principle of eliminating the periodic oscillation signal with half-wave delay by mean value restrains the oscillation errors based on forecasted time series. Compared with the damping inertial navigation system, the whole system is working in a free damping state and introduces no dynamic errors caused by maneuvers. Therefore, the method can be used for the carrier in a high-maneuver state and suppress the oscillation errors continuously. Compared with the integrated navigation system, the method has no aid of external reference information, which retains the autonomy of inertial navigation system. In a word, the new method can suppress oscillation errors in both static and maneuvering states on the condition of maintaining the autonomy of inertial navigation system, which has several advantages over the conventional methods.

The outline of the remainder of this paper is as follows. Analysis of periodic oscillation errors of SINS is given in Section 2. Method for oscillation error restriction based on forecasted time series is introduced in Section 3. Section 4 explains the method for obtaining forecasted time series by curve fitting based on least square method. Section 5 shows the simulation results and Section 6 presents the system test results. Finally, Section 7 concludes the paper.

## 2. Analysis of Periodic Oscillation Errors of SINS

In order to simplify the problem, the analysis is carried out with the error equation of SINS at rest, ignoring the vertical channel:

(1) X=FX+W

where $X={\left[\begin{array}{cccccc} \text{δ}V_{E} & \text{δ}V_{N} & \text{δφ} & \Phi_{E} & \Phi_{N} & \Phi_{U} \end{array}\right]}^{T}$,

$$F=\left[\begin{array}{cccccc} 0 & 2{\text{ω}}_{iet}^{z} & 0 & 0 & -g & 0\\ -2{\text{ω}}_{iet}^{z} & 0 & 0 & g & 0 & 0\\ 0 & -\frac{1}{R} & 0 & 0 & 0 & 0\\ 0 & -\frac{1}{R} & 0 & 0 & {\text{ω}}_{iet}^{z} & -{\text{ω}}_{iet}^{y}\\ -\frac{1}{R} & 0 & -{\text{ω}}_{iet}^{z} & -{\text{ω}}_{iet}^{z} & 0 & 0\\ \frac{1}{R}\tan\text{φ} & 0 & {\text{ω}}_{iet}^{y} & {\text{ω}}_{iet}^{y} & 0 & 0 \end{array}\right]$$

$W={\left[\begin{array}{cccccc} A_{E} & A_{N} & 0 & G_{E} & G_{N} & G_{U} \end{array}\right]}^{T}$, ${\text{ω}}_{iet}^{z}={\text{ω}}_{ie}\sin\text{φ}$; ${\text{ω}}_{ie}$ is the earth rotation rate; *R* is the radius of the earth; *g* is the acceleration of gravity; φ is the latitude; $\text{δ}V_{E}$, $\text{δ}V_{N}$ is the east velocity error and north velocity error respectively; δφ is the latitude error; $\Phi_{E}$, $\Phi_{N}$, $\Phi_{U}$ is the east, north, and up platform angle error, respectively; ${Α}_{E}$, ${Α}_{N}$ is the measurement error for east accelerometer and north accelerometer respectively; $G_{E}$, $G_{N}$, $G_{U}$ is the measurement error for east gyroscope, north gyroscope, and up gyroscope, respectively.

Doing the Laplace transform on both sides of Equation (1) and rearranging the equation gets:

(2) $$X\left(s\right)={\left(sI-F\right)}^{-1}\left(X\left(0\right)+W\left(s\right)\right)$$

Known from errors’ propagation characteristic of SINS, gyro bias drift and accelerometer bias are the main error sources, and from this we obtain:

(3) $$\{\begin{array}{lll} G_{E}\left(s\right)=\frac{{\text{ε}}_{E}}{s} & G_{N}\left(s\right)=\frac{{\text{ε}}_{N}}{s} & G_{U}\left(s\right)=\frac{{\text{ε}}_{U}}{s}\\ A_{E}\left(s\right)=\frac{\nabla_{E}}{s} & A_{N}\left(s\right)=\frac{\nabla_{N}}{s} \end{array}$$

where ${\text{ε}}_{E}$, ${\text{ε}}_{N}$, ${\text{ε}}_{U}$ is gyro bias drift for east gyroscope, north gyroscope, and up gyroscope, respectively; $\nabla_{E}$, $\nabla_{N}$ is accelerometer bias for east accelerometer and north accelerometer, respectively.

The system errors can be obtained by substituting Equation (3) into Equation (2). Taking $\Phi_{E}$ and δφ as examples, the expressions are:

(4) $$\Phi_{E}\left(s\right)=C_{42}\left(\text{δ}V_{N0}+\frac{\nabla_{N}}{s}\right)+C_{43}{\text{δφ}}_{\text{0}}+C_{44}\left(\Phi_{E0}+\frac{{\text{ε}}_{E}}{s}\right)+C_{45}\left(\Phi_{N0}+\frac{{\text{ε}}_{N}}{s}\right)+C_{46}\left(\Phi_{U0}+\frac{{\text{ε}}_{U}}{s}\right)$$

(5) $$\text{δφ}\left(s\right)=C_{32}\left(\text{δ}V_{N0}+\frac{\nabla_{N}}{s}\right)+C_{33}{\text{δφ}}_{0}+C_{34}\left(\Phi_{E0}+\frac{{\text{ε}}_{E}}{s}\right)+C_{35}\left(\Phi_{N0}+\frac{{\text{ε}}_{N}}{s}\right)+C_{36}\left(\Phi_{U0}+\frac{{\text{ε}}_{U}}{s}\right)$$

where $C_{42}=-\frac{1}{R\Delta_{1}\left(s\right)}$; $C_{43}=-\frac{{\text{ω}}_{ie}^{2}s}{\Delta_{2}\left(s\right)}$; $C_{44}=\frac{s^{3}}{\Delta_{2}\left(s\right)}$; $C_{45}=\frac{{\text{ω}}_{iet}^{z}s^{2}}{\Delta_{2}\left(s\right)}$; $C_{46}=-\frac{{\text{ω}}_{iet}^{y}s^{2}}{\Delta_{2}\left(s\right)}$; $C_{32}=\frac{1}{R\Delta_{1}\left(s\right)}$; $C_{33}=\frac{s\left(s^{2}+{\text{ω}}_{s}^{2}+{\text{ω}}_{ie}^{2}\right)}{\Delta_{2}\left(s\right)}$; $C_{34}=\frac{{\text{ω}}_{s}^{2}s}{\Delta_{2}\left(s\right)}$; $C_{35}=\frac{{\text{ω}}_{s}^{2}{\text{ω}}_{iet}^{z}}{\Delta_{2}\left(s\right)}$; $C_{36}=-\frac{{\text{ω}}_{s}^{2}{\text{ω}}_{iet}^{y}}{\Delta_{2}\left(s\right)}$.

where $\Delta_{1}\left(s\right)=\left(s^{2}+{\text{ω}}_{s}^{2}\right)$; $\Delta_{2}\left(s\right)=\left(s^{2}+{\text{ω}}_{ie}^{2}\right)\left(s^{2}+{\text{ω}}_{s}^{2}\right)$; ${\text{ω}}_{s}$ is the Schuler oscillation frequency; ${\text{ω}}_{ie}$ is the Earth oscillation frequency; $\Phi_{E0}$, $\Phi_{N0}$, $\Phi_{U0}$ are the initial values of platform angle errors; ${\text{δφ}}_{0}$ is the initial value of latitude error; $\text{δ}V_{N0}$ is the initial value of north velocity error.

Applying the inverse Laplace transform on Equations (4) and (5), considering ${\text{ω}}_{s}>>{\text{ω}}_{ie}$:

(6) $$\Phi_{E}\left(t\right)=M_{1}\sin{\text{ω}}_{s}t+M_{2}\cos{\text{ω}}_{s}t+M_{3}\sin{\text{ω}}_{ie}t+M_{4}\cos{\text{ω}}_{ie}t+M_{5}$$

(7) $$\text{δφ}\left(t\right)=L_{1}\sin{\text{ω}}_{s}t+L_{2}\cos{\text{ω}}_{s}t+L_{3}\sin{\text{ω}}_{ie}t+L_{4}\cos{\text{ω}}_{ie}t+L_{5}$$

where $M_{1}=\frac{1}{{\text{ω}}_{s}}{\text{ε}}_{E}-\frac{1}{R{\text{ω}}_{s}}\text{δ}V_{N0}$; $M_{2}=-\frac{{\text{ω}}_{iet}^{z}}{{\text{ω}}_{s}^{2}}{\text{ε}}_{N}+\frac{{\text{ω}}_{iet}^{y}}{{\text{ω}}_{s}^{2}}{\text{ε}}_{U}+\Phi_{E0}+\frac{1}{R{\text{ω}}_{s}^{2}}\nabla_{N}$; $M_{3}=-\frac{{\text{ω}}_{ie}}{{\text{ω}}_{s}^{2}}{\text{ε}}_{E}$; $M_{4}=\frac{{\text{ω}}_{iet}^{z}}{{\text{ω}}_{s}^{2}}{\text{ε}}_{N}-\frac{{\text{ω}}_{iet}^{y}}{{\text{ω}}_{s}^{2}}{\text{ε}}_{U}-\Phi_{E0}$; $M_{5}=-\frac{1}{R{\text{ω}}_{s}^{2}}\nabla_{N}$; $L_{1}=-\frac{1}{{\text{ω}}_{s}}{\text{ε}}_{E}+\frac{1}{R{\text{ω}}_{s}}\text{δ}V_{N0}$; $L_{2}=\frac{{\text{ω}}_{iet}^{z}}{{\text{ω}}_{s}^{2}}{\text{ε}}_{N}-\frac{{\text{ω}}_{iet}^{y}}{{\text{ω}}_{s}^{2}}{\text{ε}}_{U}-\Phi_{E0}-\frac{1}{R{\text{ω}}_{s}^{2}}\nabla_{N}$; $L_{3}=\frac{1}{{\text{ω}}_{ie}}{\text{ε}}_{E}+\sin\text{φ}\Phi_{N0}-\cos\text{φ}\Phi_{U0}$; $L_{4}=\frac{\sin\text{φ}}{{\text{ω}}_{ie}}{\text{ε}}_{N}+\frac{\cos\text{φ}}{{\text{ω}}_{ie}}{\text{ε}}_{U}+\Phi_{E0}+{\text{δφ}}_{0}$; $L_{5}=+\frac{\sin\text{φ}}{{\text{ω}}_{ie}}{\text{ε}}_{E}-\frac{\cos\text{φ}}{{\text{ω}}_{ie}}{\text{ε}}_{U}+\frac{\nabla_{N}}{g}$.

Equations (6) and (7) show that although the steady-state values of $\Phi_{E}$ and δφ are constant, Schuler and Earth periodic oscillation errors are contained in $\Phi_{E}$ and δφ. From the point of mathematics, these oscillation errors are the summation of a number of sine and cosine functions, whose oscillation frequency is ${\text{ω}}_{s}$ or ${\text{ω}}_{ie}$ and the amplitudes of periodic functions are decided by the corresponding error sources. According to Equation (2), other SINS errors contain the similar periodic oscillation errors, which greatly reduce the precision of inertial navigation system due to having the same characteristic root.

The conclusion above is given by ignoring Foucault periodic oscillation errors, which influences the result little since Foucault periodic oscillation errors only modulate Schuler periodic oscillation errors.

## 3. Method for Oscillation Errors Restriction Based on Forecasted Time Series

### 3.1. Principle of Eliminating Periodic Oscillation Signal with Half-Wave Delay by Mean Value

A periodic oscillation signal whose cycle is *T* can be written as:

(8) $$y_{1}\left(t\right)=A\sin\text{ω}t$$

where ω=2π/T.

Another periodic oscillation signal with cycle *T* is:

(9) $$y_{2}\left(t\right)=A\sin\text{ω}\left(t+\frac{T}{2}\right)=A\sin\left(\text{ω}t+\pi \right)=-A\sin\text{ω}t$$

Averaging Equations (8) and (9) gives:

(10) $$y\left(t\right)=\frac{y_{1}\left(t\right)+y_{2}\left(t\right)}{2}=\frac{A\sin\text{ω}t-A\sin\text{ω}t}{2}=0$$

Concluded from Equation (10), the oscillation can be suppressed by the mean of two signals when their phases vary by π or their amplitudes are the opposite of each other. So the Schuler and Earth periodic oscillation errors can be eliminated if the difference between two phases equals to π.

### 3.2. Method for Oscillation Errors Restriction Based on Forecasted Time Series

Inertial navigation systems cannot switch into navigation mode until the alignment process is done. Whether using independent initial alignment or integrated initial alignment, errors of inertial devices directly determine the precision of initial alignment of inertial navigation system. Different from physical closed-loop control of platform inertial navigation system, SINS allows executing multiple sets of navigation algorithms at the same time.

SINS performs its initial alignment process in the stationary state. The theoretical values the IMU senses are fixed, so the measurement data from the IMU can be regarded as a stationary time series containing random noise. Therefore, the prediction values of time series can be obtained by analyzing and processing the measurement data from the IMU in the process of initial alignment.

In order to compensate for oscillation errors with cycle *T*, prospective time series whose time length is *T/2* is needed. When the error of the static initial alignment process satisfies the requirements, the initial alignment process is not finished right away and an auxiliary navigation algorithm is executed, using the result of initial alignment process at current moment *t* and prospective time series. Auxiliary navigation algorithm does not turn into a real-time navigation state until the prospective time series is used up, while the main navigation algorithm starts working with the result of initial alignment process at current moment t′.

To compensate oscillation errors with cycle *T*, making use of forecasted time series lets the phases of oscillation errors in auxiliary navigation algorithm differ from the ones of the main navigation algorithm by π. The process diagram is as follow.

**Figure 2:**
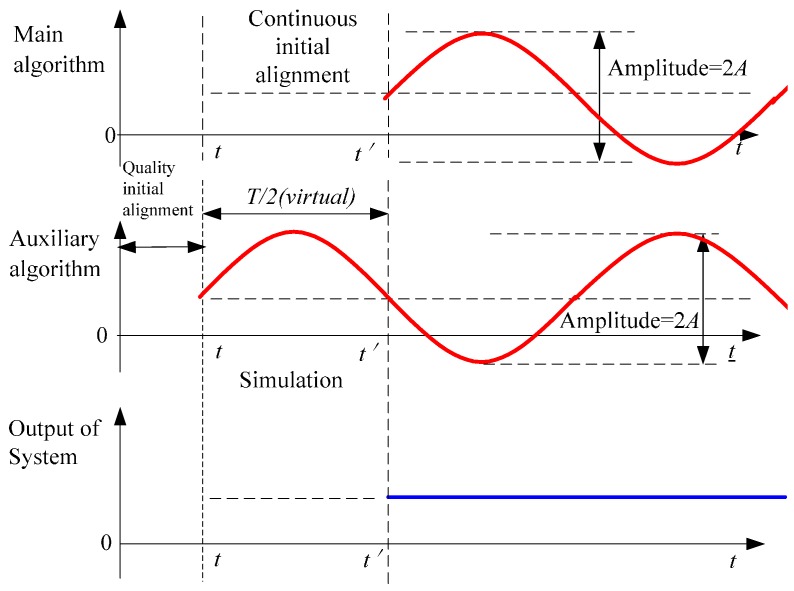
Principle diagram of suppressing oscillation errors based on forecasted time series.

As shown in Figure 2, making use of forecasted time series obtained from the static initial alignment process lets the phases of oscillation errors with cycle *T* in the auxiliary navigation algorithm differ from the ones of the main navigation algorithm by π. The simulation consumes very little time because it is not a real-time process. In other words, oscillation errors are restricted with little cost.

Based on the process shown in Figure 2, reanalyzing and arranging Equations (5) and (6) gives:

(11) $$\Phi_{E}\left(t\right)=\Phi_{E1}\left(t\right)+\Phi_{E2}\left(t\right)+M_{5}$$

(12) $$\text{δφ}\left(t\right)={\text{δφ}}_{1}\left(t\right)+{\mathrm{dj}}_{2}\left(t\right)+L_{5}$$

where $\Phi_{E1}\left(t\right)=M_{1}\sin{\text{ω}}_{s}t+M_{2}\cos{\text{ω}}_{s}t$; $\Phi_{E2}\left(t\right)=M_{3}\sin{\text{ω}}_{ie}t+M_{4}\cos{\text{ω}}_{ie}t$; ${\text{δφ}}_{1}\left(t\right)=L_{1}\sin{\text{ω}}_{s}t+L_{2}\cos{\text{ω}}_{s}t$; ${\text{δφ}}_{2}\left(t\right)=L_{3}\sin{\text{ω}}_{ie}t+L_{4}\cos{\text{ω}}_{ie}t$.

Considering $T=2\text{π}/{\text{ω}}_{s}$, averaging the system errors of the auxiliary navigation algorithm and main navigation algorithm presents:

$$\begin{array}{l} {\tilde{\Phi }}_{E}\left(t\right)=\frac{1}{2}\left[\Phi_{E}\left(t\right)+\Phi_{E}\left(t+\frac{T}{2}\right)\right]=\frac{1}{2}\left[\Phi_{E1}\left(t\right)+\Phi_{E2}\left(t\right)+\Phi_{E1}\left(t+\frac{T}{2}\right)+\Phi_{E2}\left(t+\frac{T}{2}\right)\right]+M_{5}\\ =\frac{1}{2}[M_{1}\sin{\text{ω}}_{s}t+M_{2}\cos{\text{ω}}_{s}t+M_{1}\sin\left({\text{ω}}_{s}t+\text{π}\right)+M_{2}\cos\left({\text{ω}}_{s}t+\text{π}\right)+M_{3}\sin{\text{ω}}_{ie}t+M_{4}\cos{\text{ω}}_{ie}t\\ +M_{3}\sin\left({\text{ω}}_{ie}t+\frac{{\text{ω}}_{ie}\text{π}}{{\text{ω}}_{s}}\right)+M_{4}\cos\left({\text{ω}}_{ie}t+\frac{{\text{ω}}_{ie}\text{π}}{\omega_{s}}\right)]+M_{5} \end{array}$$

According to the trigonometric function relationship, simplifying the mean shows:

(13) $${\tilde{\Phi }}_{E}\left(t\right)=\frac{1}{2}\left[\Phi_{E2}\left(t\right)+\Phi_{E2}\left(t+\frac{T}{2}\right)\right]+M_{5}$$

Likewise:

(14) $$\text{δ}\tilde{\text{φ}}\left(t\right)=\frac{1}{2}\left[{\text{δφ}}_{2}\left(t\right)+{\text{δφ}}_{2}\left(t+\frac{T}{2}\right)\right]+L_{5}$$

Equations (13) and (14) show that when $T=2\text{π}/{\text{ω}}_{s}$, Schuler periodic oscillation errors can be eliminated directly. Owing to the fact that Foucault periodic oscillation errors modulate Schuler periodic oscillation errors, Foucault periodic oscillation errors can be compensated as well.

Assuming $T=2\text{π}/{\text{ω}}_{ie}$, averaging system errors of auxiliary navigation algorithm and main navigation algorithm gives:

(15) $$\text{δ}\bar{\text{φ}}\left(t\right)=\frac{1}{2}\left[\text{δφ}\left(t\right)+\text{δφ}\left(t+\frac{T}{2}\right)\right]=\frac{1}{2}\left[{\text{δφ}}_{1}\left(t\right)+{\text{δφ}}_{1}\left(t+\frac{T}{2}\right)\right]+L_{5}$$

Likewise:

(16) $${\bar{\Phi }}_{E}\left(t\right)=\frac{1}{2}\left[\Phi_{E1}\left(t\right)+\Phi_{E1}\left(t+\frac{T}{2}\right)\right]+M_{5}$$

Known from Equations (15) and (16), Earth periodic oscillation errors can be compensated when $T=2\text{π}/{\text{ω}}_{ie}$.

Among the errors of SINS, Earth periodic oscillation errors are obvious in yaw error and latitude error; horizontal attitude and horizontal velocity are influenced by Schuler periodic oscillation errors and Foucault periodic oscillation errors. Therefore, according to the different forms of oscillation errors, making use of forecasted time series with different time length can suppress Schuler, Foucault, and Earth periodic oscillation errors at the same time.

The main navigation algorithm outputs the navigation parameters noted as $B_{i}$, the first auxiliary navigation algorithm using forecasted time series with time length of $\text{π}/{\text{ω}}_{s}$ outputs the navigation parameters noted as $C_{i}$, and the second auxiliary navigation algorithm using forecasted time series with time length of $\text{π}/{\text{ω}}_{ie}$ outputs the navigation parameters noted as $D_{i}$. Schuler periodic oscillation errors and Foucault periodic oscillation errors of horizontal attitude and horizontal velocity in $B_{i}$ can be compensated by the ones in $C_{i}$ , and Earth periodic oscillation errors of position and yaw in $B_{i}$ can be compensated by the ones in $D_{i}$. The formulation is as follow.

(17) $$\begin{array}{cc} \{\begin{array}{l} {S^{\prime }}_{i}=\left(B_{i}+C_{i}\right)/2\\ {S^{\prime \prime }}_{i}=\left(B_{i}+D_{i}\right)/2 \end{array} & i=1,2,...,7 \end{array}$$

Known from Equation (17), the method for oscillation error restriction based on forecasted time series introduces no external reference information and retains autonomy of the inertial navigation system. Because the inertial navigation system is still working without a damping state, there are no dynamic errors encouraged by the acceleration from maneuvers.

The method is suitable for SINS whose initial alignment process is finished in a stationary state. It is worth noting that during the movement of carrier ${\text{ε}}_{E}$, ${\text{ε}}_{N}$, ${\text{ε}}_{U}$, $\nabla_{E}$, $\nabla_{N}$ would vary over time due to the factors, such as changes of the attitude, which weakens the effect of suppressing oscillation error caused by them. However, the initial errors are constant and so oscillation errors caused by them can be restrained effectively all the time.

Although the results are given by the analysis of the error equation of SINS at rest, they are also applicable to SINS in a motion state. As the error equation of SINS in motion state is too complicated to obtain the analytical solution, it is very difficult to give a detailed deduction and some qualitative analyses would be done here. For the carrier with velocity in the range of 0–50 m/s, $\frac{V_{E}}{R}$ and $\frac{V_{N}}{R}$ are at least one order of magnitude smaller than ${\text{ω}}_{ie}$, in general. Thus, the difference between the static state and motion state is small and the error equation of SINS at rest can be used for concise analyses in this condition. For the carrier with high velocity, Schuler oscillation period and Earth oscillation period would change over time. However, in the static state, obtaining a set of navigation solution whose phases of oscillation errors vary by π is equal to getting a set of navigation solution whose amplitudes of oscillation errors are the opposite of each other in the beginning. No matter what the subsequent movement state is, the frequencies of the oscillation errors are always the same. Thus, the effect of suppressing oscillation errors always exists, which will be analyzed and confirmed in the subsequent simulation.

## 4. Method for Obtaining Forecasted Time Series by Curve Fitting Based on Least Square Method

### 4.1. Curve Fitting Based on Least Square Method

A set of data for a certain physical process is known as:

(18) $$\left(x_{i},f\left(x_{i}\right)\right),i=1,2,\cdots ,m$$

Seeking an approximate function ϕ(x) of $f(x_{i})$ in a particular class of functions φ(x), their residual on $x_{i}$ is:

(19) $${\text{δ}}_{i}=\phi \left(x_{i}\right)-f\left(x_{i}\right),i=1,2,\cdots ,m$$

Minimizing the residual according to some metric which is always chosen as:

(20) $${\left\|\text{δ}\right\|}_{2}=(\sum_{i=0}^{m}{\text{δ}}_{i}^{2}{)}^{\frac{1}{2}}=\{\sum_{i=0}^{m}{\left[\phi (x_{i})-f(x_{i})\right]}^{2}{\}}^{\frac{1}{2}}$$

Or:

(21) $${\left\|\text{δ}\right\|}_{2}^{2}=\sum_{i=0}^{m}{\text{δ}}_{i}^{2}=\sum_{i=0}^{m}{\left[\phi (x_{i})-f(x_{i})\right]}^{2}$$

Curve fitting based on least square method makes the sum of error squares minimal and gives the optimal estimation in minimum variance criterion.

### 4.2. Problem Description

Curve fitting based on least square method can objectively predict the time series with arbitrary length on the ideal condition, where the carrier is in the stationary state. However, due to the presence of various disturbances, the carrier may have small angular motions in the actual application. Several forms of the interference of small angular motions in the output of gyro are shown as follow.

**Figure 3:**
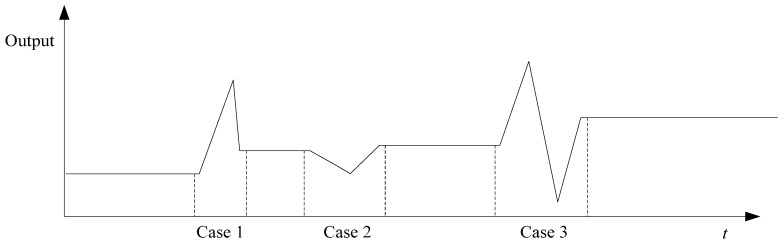
Several forms of the interference of small angular motions in the output of gyro.

Case 1: IMU moves by a small angular along positive direction (clockwise);

Case 2: IMU moves by a small angular along negative direction (anti-clockwise);

Case 3: IMU moves by a small angular along positive direction and then moves along negative direction.

Figure 3 shows that small angular motions bring a certain bias to forecasted time series obtained by curve fitting based on least square method. Therefore, it is necessary to distinguish small angular motions from random noise and remove the effect of small angular motions on forecasted time series.

### 4.3. Forecasted Time series Obtained by Curve Fitting Based on Least Square Method

Measurement time series from IMU in the initial alignment process is:

(22) $$Z\left(t\right)={\left[{\text{ω}}_{ib}^{b}\left(t\right),f_{b}\left(t\right)\right]}^{T}$$

where ${\text{ω}}_{ib}^{b}\left(t\right)={\left[{\text{ω}}_{x}\left(t\right),{\text{ω}}_{y}\left(t\right),{\text{ω}}_{z}\left(t\right)\right]}^{T}$; $f_{b}\left(t\right)={\left[f_{x}\left(t\right),f_{y}\left(t\right),f_{z}\left(t\right)\right]}^{T}$; ${\text{ω}}_{x}\left(t\right)$, ${\text{ω}}_{y}\left(t\right)$, ${\text{ω}}_{z}\left(t\right)$ is the measurement time series for gyroscope in *x* axis, gyroscope in *y* axis, and gyroscope in *z* axis, respectively. $f_{x}\left(t\right)$ , $f_{y}\left(t\right)$, $f_{z}\left(t\right)$ is the measurement time series for accelerometer in *x* axis, accelerometer in *y* axis, and accelerometer in *z* axis, respectively.

Measurement time series for gyroscopes:

(23) $${\text{ω}}_{ib}^{b}\left(t\right)={\text{ω}}_{ie}^{b}\left(t\right)+{\text{ω}}_{ep}^{b}\left(t\right)+{\text{ω}}_{pb}^{b}\left(t\right)$$

Integrating on both sides gives:

(24) $$\int {\text{ω}}_{ib}^{b}\left(t\right)dt=\int {\text{ω}}_{ie}^{b}\left(t\right)dt+\int {\text{ω}}_{ep}^{b}\left(t\right)dt+\int {\text{ω}}_{pb}^{b}\left(t\right)dt$$

Because the carrier is static, the theoretical value of $\omega_{ep}^{b}\left(t\right)$ is zero. Assuming the carrier have small angular motion during the time of $t_{1}∼t_{2}$:

(25) $$\int_{0}^{t}{\text{ω}}_{ib}^{b}\left(t\right)dt=\int_{0}^{t}{\text{ω}}_{ie}^{b}\left(t\right)dt+\int_{0}^{t}{\text{ω}}_{pb}^{b}\left(t\right)dt=\int_{0}^{t}{\text{ω}}_{ie}^{b}\left(t\right)dt+\int_{t_{1}}^{t_{2}}{\text{ω}}_{pb}^{b}\left(t\right)dt$$

where $\int_{0}^{t}{\text{ω}}_{ib}^{b}\left(t\right)dt$ is the variation of angular of body frame, with respect to inertial frame in the body frame. Integral operation can eliminate the interference of noise with high frequency in ${\text{ω}}_{ib}^{b}\left(t\right)$. ${\text{ω}}_{ie}^{b}\left(t\right)$ is the projection of ${\text{ω}}_{ie}$ whose magnitude is 10_−5_ in body frame, so ${\text{ω}}_{pb}^{b}\left(t\right)$ produced by a small angular motion is higher by several orders of magnitude, in general. Therefore, $\int_{0}^{t}{\text{ω}}_{ib}^{b}\left(t\right)dt$ changes significantly during t_1_~t_2_.

**Figure 4:**
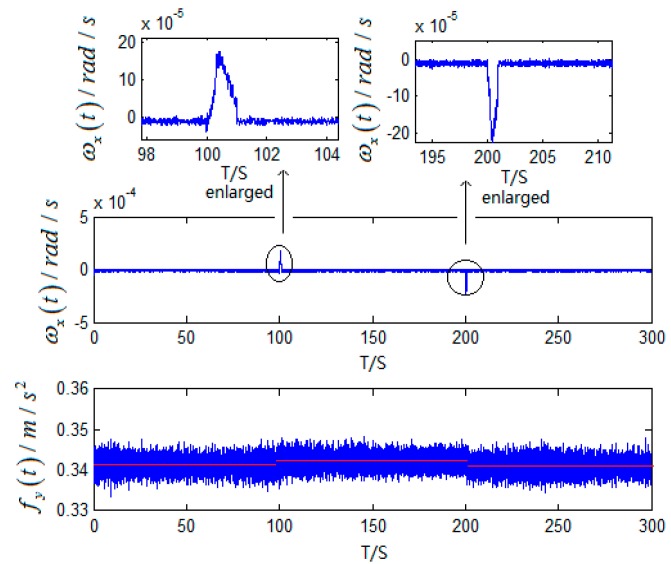
The outputs of gyro and accelerometer with the interference of small angular motions.

In the stationary state, the outputs of gyro and accelerometer in fiber gyroscope inertial navigation systems with the interference of small angular motions are as follows in Figure 4.

**Figure 5:**
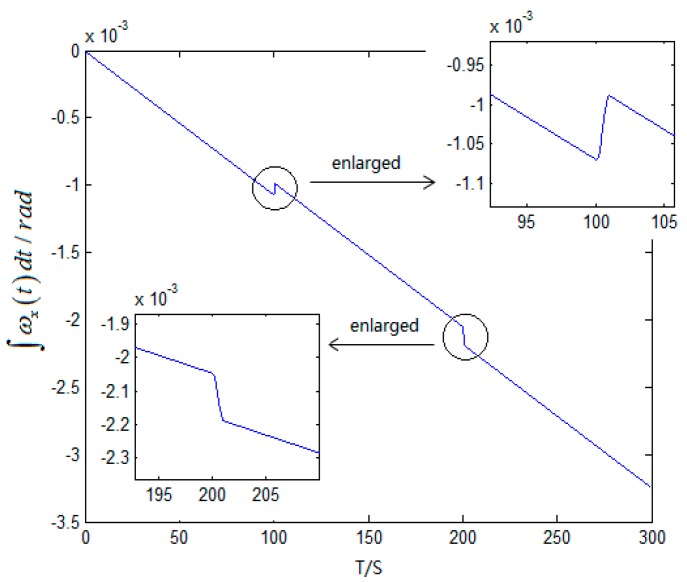
Variation of $\int_{0}^{t}{\text{ω}}_{x}\left(t\right)dt$.

Known from Figure 5, angular movements of the IMU happened during 100 s~105 s and 198 s~203 s. Therefore, the time when the angular motions occurred can be distinguished by variation of $\int_{0}^{t}{\text{ω}}_{ib}^{b}\left(t\right)dt$*.*

Based on the output of gyro shown in Figure 4, the variation of $\int_{0}^{t}{\text{ω}}_{x}\left(t\right)dt$ is:

Assuming small angular motion occurred more than one time, making use of variation of $\int_{0}^{t}{\text{ω}}_{ib}^{b}\left(t\right)dt$ determines the time when small angular motions happened. Z(t) is divided into *N* time series, noted as $Z_{i}\left(t\right)$ by determined span, doing curve fitting based on least square method on every time series gives estimations ${\hat{E}}_{i}\left(t\right)$ for each time series where i=1,2,⋯,N. Get ${\hat{Z}}_{M}\left(t\right)$ by the formulation:

(26) $${\hat{Z}}_{M}\left(t\right)=Z_{M}\left(t\right)+{\hat{E}}_{N}\left(t\right)-{\hat{E}}_{M}\left(t\right),M=1,2,\cdots ,N$$

And the new time series is:

(27) $$\hat{Z}\left(t\right)=\left[{\hat{Z}}_{1}\left(t\right),{\hat{Z}}_{2}\left(t\right),\cdots ,{\hat{Z}}_{N}\left(t\right)\right]$$

$\hat{Z}\left(t\right)$ is a new time series gained by eliminating angular motions in Z(t). Processing ${\text{ω}}_{x}\left(t\right)$ and $f_{y}\left(t\right)$ shown in Figure 4 obtains the new time series and curve fitting as follows.

**Figure 6:**
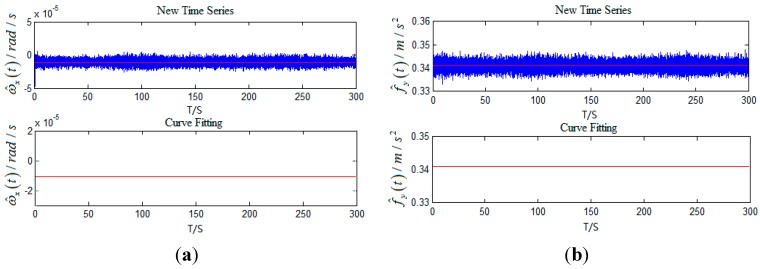
(**a**) The new time series and curve fitting of gyro; (**b**) The new time series and curve fitting of accelerometer.

According to Figure 6, curve fitting based on least square for $\hat{Z}\left(t\right)$ can objectively predict the time series with arbitrary length in the future. Since the least squares method is a type of unbiased estimator, forecasted time series still contains gyro bias drift and accelerometer bias. Schuler and Foucault periodic oscillation errors can be restrained with forecasted time series for $\text{π}/{\text{ω}}_{s}$ and Earth periodic oscillation errors can be suppressed with forecasting time series for $\text{π}/{\text{ω}}_{ie}$.

## 5. Computer Simulations

### 5.1. Simulation of Maneuvering Carrier

#### 5.1.1. Simulation with No Inertia Device Errors

(1) Simulation conditions

There are no inertia device errors.

The initial alignment errors: Errors of initial position: longitude 2″, latitude 2″; Errors of initial velocity: east velocity 0.1 m/s, north velocity 0.1 m/s; Errors of initial attitude: pitch 0.6′, roll 0.6′, yaw 7′.

Others: Time of initial alignment in static state: 10 min; Time of simulation: 24 h; Step of simulation: 0.01 s; After the whole system turns into navigation mode, the range of east velocity is in 0~300 m/s, the range of north velocity is in 0~30 m/s, the range of attitude components is in 0°~90°, the change rate of velocity is as follow.

**Figure 7:**
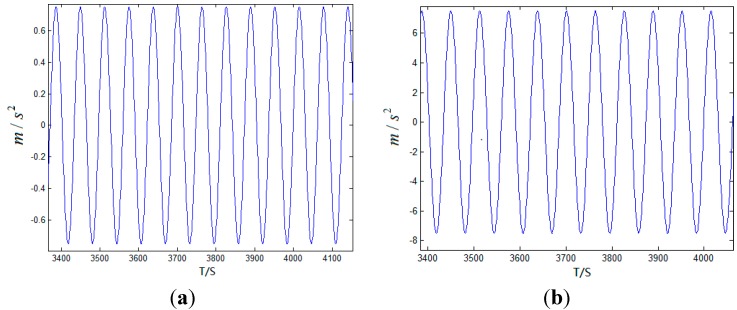
(**a**) The change rate of north velocity; (**b**) The change rate of east velocity.

In the whole simulation time, the change rate of velocity varies over time, as shown in Figure 7, and the traditional damping technology without using external reference information cannot apply to this condition.

(2) Simulation results

Based on the conditions above, the simulation results are as follows.

**Figure 8:**
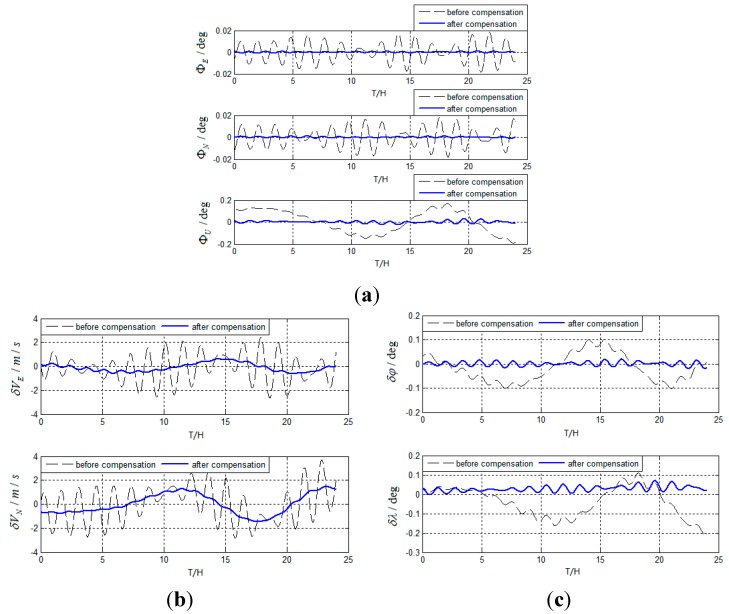
(**a**) Simulation results of attitude errors for maneuvering carrier with no inertia device errors; (**b**) Simulation results of velocity errors for maneuvering the carrier with no inertia device errors; (**c**) Simulation results of position errors for maneuvering the carrier with no inertia device errors.

Figure 8 shows that although the carrier is in the maneuvering state sustainably, Schuler, Foucault, and Earth periodic oscillation errors caused by initial errors can be suppressed, which is impossible for the damping inertial navigation system. Known from the simulation curves of $\Phi_{U}$, δφ, and δλ, the period of oscillation errors that relate to the rotation of the Earth is smaller than 24 h, primarily due to the high velocity. However, the method has good performance in restricting the oscillation errors still, which indicates that the method is not affected by the motion state of the carrier.

#### 5.1.2. Simulation with Inertia Device Errors

(1) Simulation conditions

Errors of IMU: Gyro bias drift: 0.01°/h; Angle random walk: 0.005°/h; Accelerometer bias drift: 1 × 10^−4^ g; Velocity random walk: 5 × 10^−4^ g.

The other conditions are the same as the simulation with no inertia device errors.

(2) Simulation results

Based on the conditions above, the simulation results are as follows.

**Figure 9:**
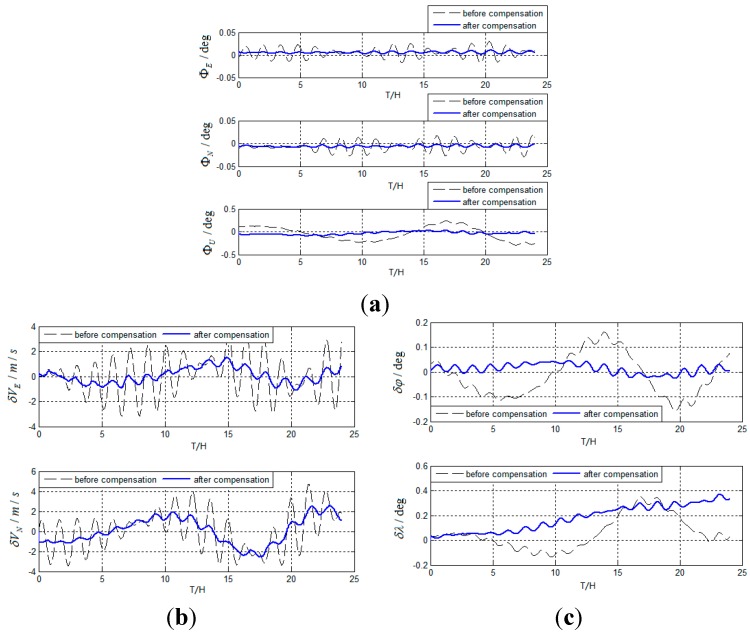
(**a**) Simulation results of attitude errors for maneuvering the carrier with inertia device errors; (**b**) Simulation results of velocity errors for maneuvering the carrier with inertia device errors; (**c**) Simulation results of position errors for maneuvering the carrier with inertia device errors.

Known from Figure 9, although ${\text{ε}}_{E}$, ${\text{ε}}_{N}$, ${\text{ε}}_{U}$, $\nabla_{E}$, $\nabla_{N}$ vary over time, the new method has good performance in suppressing Schuler, Foucault, and Earth periodic oscillation errors as well, and greatly improves the precision of SINS. 

## 6. System Test

### 6.1. Test Equipment

**Figure 10:**
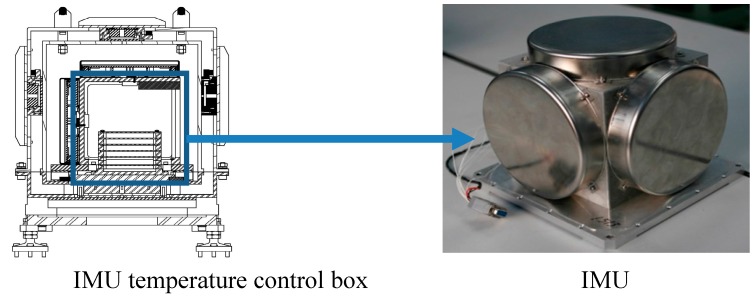
Fiber gyroscope inertial navigation system.

Using the fiber gyroscope inertial navigation system shown in Figure 10 validates the method for oscillation error restriction based on forecasted time series.

As shown in Figure 10, the fiber gyroscope inertial navigation system is accompanied with a temperature control box. The IMU is put in the temperature control box in order to insulate external interference from thermal and magnetic sources. Some main performance parameters of the IMU are as follows.

Gyros: Gyro bias drift <0.005°/h; Angle random walk <0.005°/h; Scale factor non-linearity 10 ppm; Scale factor sign asymmetry 10 ppm; Scale factor reiteration 10 ppm. Accelerometers: Accelerometer bias drift <7 × 10^−5^ g; Velocity random walk <5 × 10^−5^ g. Scale factor 1.3~1.6 mA/g; two order nonlinear coefficient <µg/g^2^.

Reference datum is a high-precision, three-axis turntable (SGT-3). Its performance parameters are as follows. Wobble error: ±2″; Axis perpendicularity: ±2″; Positioning accuracy: ±3″; Position control resolution: 0.0001°; Position repeatability accuracy: ±1″; Rate resolution: 0.0001°/s. Owing to the high attitude measurement accuracy of the turntable, it can be used as the attitude reference. Since the IMU is placed in the swing center, the velocity of the IMU is approximately zero and the position is unchanged.

**Figure 11:**
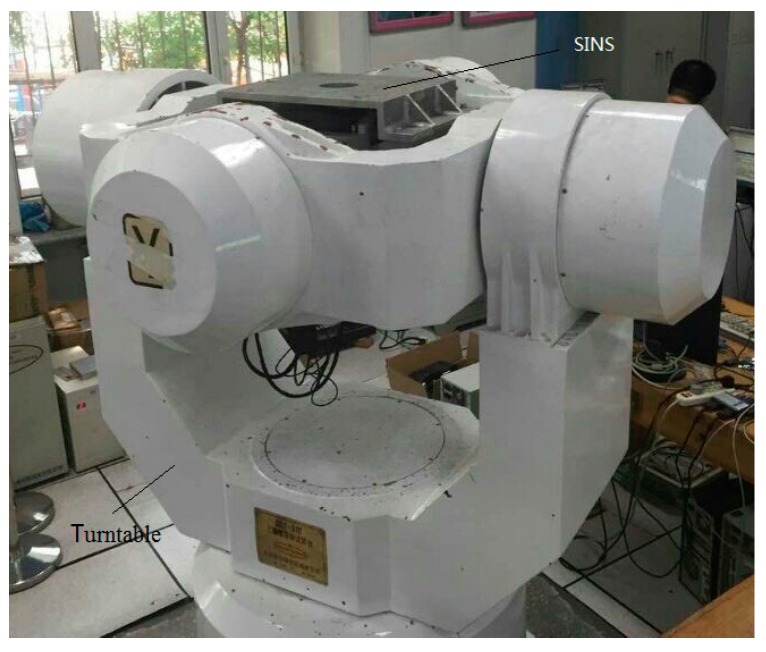
High-precision, three-axis turntable and SINS.

The test equipment is shown in Figure 11.

### 6.2. Test

#### 6.2.1. Test Preparation

The IMU was installed on the turntable and the coordinate system of the IMU was made coincident with the turntable as closely as possible. The misalignment matrix between the two coordinate systems was obtained though calibration, which was used for error compensation.

#### 6.2.2. Test Process

(1) In order to make the outputs of gyroscopes and accelerometers stable, the temperature control system needed to make the temperature of IMU stable at 33° C. The whole process, called system startup, consumed about 1 h.

(2) After the system startup, the initial alignment process was conducted for 10 min, during which the initial values of the main algorithm and auxiliary algorithms were obtained according to the method mentioned above. Small angular motions were added in the initial alignment process with the help of the turntable.

(3) The whole system then switched to the navigation state and was tested for 25 h.

#### 6.2.3. Test Set

The turntable did not start to swing until the whole system switched to the navigation state. Swing range of the turntable was 5° and swing period of the turntable was 20 s.

#### 6.2.4. Sampling Frequency and Update Frequency 

The sampling frequency of the IMU was 100 Hz and the update frequency of the algorithms was 50 Hz.

#### 6.2.5. Reference Datum

In the process of navigation, the measurement data from the turntable was obtained at the same time. The attitude reference was chosen as the attitude measured by the turntable. The velocity reference was chosen as zero. The position reference was chosen as the local location (longitude: 126.6773°E, latitude: 45.7796°N).

#### 6.2.6. Initial Attitude Errors

Pitch: about 0.15; roll: about 0.14; yaw: about 1.3′.

### 6.3. Test Results

**Figure 12:**
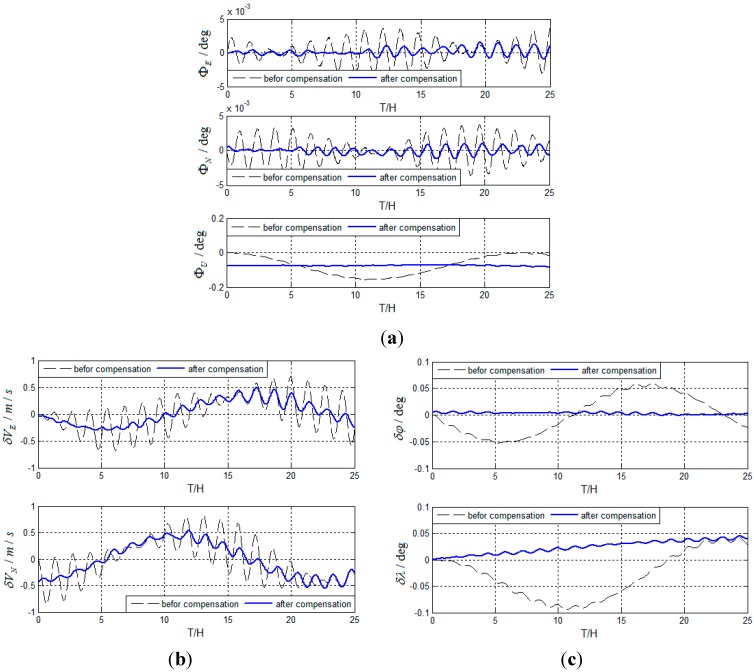
(**a**) Test results of attitude errors for actual system; (**b**) Test results of velocity errors for actual system; (**c**) Test results of position errors for actual system.

Based on actual test, test results are obtained as follows in Figure 12.

As can be seen from the simulation and test results, the method can suppress Schuler periodic oscillation errors, Foucault periodic oscillation errors, and Earth periodic oscillation errors effectively. The method can be used for the carrier in a high-maneuver state and restrain the oscillation errors continuously. The main reasons why Schuler period oscillation errors are not completely suppressed are as follows: (1) ${\text{ε}}_{E}$, ${\text{ε}}_{N}$, ${\text{ε}}_{U}$, $\nabla_{E}$, $\nabla_{N}$ vary over time; and (2) Schuler periodic oscillation errors are modulated by Foucault periodic oscillation errors, which are not compensated at all.

## 7. Conclusions

Aiming at the problem that the precision of SINS is influenced a lot by periodic oscillation errors, a method for oscillation errors restriction based on forecasted time series is proposed in this paper. Simulation and experimental results show that the method can effectively suppress periodic oscillation errors and greatly improve the precision of SINS. The method not only preserves autonomy of SINS but also introduces no dynamic errors caused by acceleration, which has important practical application value.
